# Selection of Excipients for the Preparation of Vancomycin-Loaded Poly(D,L-lactide-co-glycolide) Microparticles with Extended Release by Emulsion Spray Drying

**DOI:** 10.3390/pharmaceutics15102438

**Published:** 2023-10-09

**Authors:** Ana Jurić Simčić, Iva Erak, Biserka Cetina Čižmek, Anita Hafner, Jelena Filipović-Grčić

**Affiliations:** 1 R&D, PLIVA Croatia Ltd., TEVA Group Member, 10000 Zagreb, Croatia; ana.juricsimcic@pliva.com (A.J.S.); iva.erak@pliva.com (I.E.); biserka.cetina-cizmek@pliva.com (B.C.Č.); 2 Faculty of Pharmacy and Biochemistry, University of Zagreb, 10000 Zagreb, Croatia; ahafner@pharma.unizg.hr

**Keywords:** PLGA, microparticles, emulsions, spray drying, stabilisers

## Abstract

The aim of this study was to relate the composition of the W/O emulsion used as a starting fluid in the spray-drying process to the quality of the dry polymer particles obtained in terms of physical–chemical properties, compatibility and drug release performance. Four W/O emulsions containing vancomycin hydrochloride (VAN), an encapsulating PLGA polymer and Poloxamer^®^ 407, chitosan and/or sorbitan monooleate as stabilisers were spray-dried using an ultrasonic atomising nozzle. The microparticles obtained were micron-sized, with a volume mean diameter between 43.2 ± 0.3 and 64.0 ± 12.6 µm, and spherical with a mostly smooth, non-porous surface and with high drug loading (between 14.5 ± 0.6 and 17.1 ± 1.9% *w*/*w*). All formulations showed a prolonged and biphasic VAN release profile, with diffusion being the primary release mechanism. Microparticles prepared from the emulsions with Poloxamer^®^ 407 and sorbitan monooleate released VAN rapidly and completely within one day. The release of VAN from microparticles prepared from the emulsion without additives or with chitosan in the inner aqueous phase was significantly decreased; after four days, a cumulative release of 65% and 61%, respectively, was achieved. Microparticles with encapsulated chitosan had the largest mean particle diameter and the slowest release of VAN.

## 1. Introduction

The spray drying of a water-in-oil emulsion is a one-step, continuous microencapsulation process that enables the preparation of sustained-release microparticle systems for hydrophilic drugs with stable pharmacokinetic profiles over a long period of time. A water-in-oil emulsion is prepared by dispersing an aqueous phase containing a hydrophilic drug under high shear forces in an organic phase containing an encapsulating polymer. The resulting emulsion is passed through a set of tubes and an atomiser nozzle into a drying chamber, where the solvent evaporates and the liquid droplets are converted into a dry powder product [1]. A copolymer of lactic and glycolic acid (poly(D,L-lactide-co-glycolide); PLGA) is widely used as the encapsulation polymer due to its biocompatibility, biodegradability and non-toxicity [2]. To date, PLGA-based particle systems have been investigated for the delivery of various biopharmaceutical agents via injection routes, and several products are already on the market [3].

The microstructure of a W/O emulsion is reflected in the properties of the final polymeric microparticles. The deformation and break-up of the droplets of the dispersed phase can be caused by the processing conditions applied and can alter the microstructure of the original emulsion. This impacts the drug loading, encapsulation efficiency, morphology and drug release of the drug-loaded polymeric microparticles that are the end product of spray drying.

Other than influencing the stability and rheology of the initial WO emulsion, surfactants and hydrophilic polymers can also stabilise the air–liquid interfaces to which large molecules irreversibly attach, preventing their physical degradation. In addition, the dehydration that occurs during spray drying can lead to structural changes and the denaturation of proteins, as water maintains their structural stability and preserves the thermodynamics of the hydrogen bonds between enzymes and substrates [4,5]. In general, the co-encapsulation of a hydrophilic polymer forms a viscous microenvironment and minimises the negative influence of the organic solvent on the integrity of the protein, as suggested in several publications [6,7]. In addition, poloxamers form a micellar structure that mechanically entraps water at elevated temperatures, replacing the intermolecular interactions of water molecules removed by the drying process [8,9,10]. Finally, formulation adjuvants such as surfactants can improve the yield and processability of spray-dried powders and promote their dispersibility and reconstitution [4,11].

The choice of the encapsulating polymer depends on several factors, such as the route of administration, the drug loading of the microparticles, the rate of release to achieve the therapeutic concentration and the polymer degradation rate [12]. PLGA is usually described by the lactide-to-glycolide (L/G) ratio, molecular weight, intrinsic viscosity and end group (acid or ester). These factors influence the encapsulation efficiency and the rate of degradation of the polymer chain. Polymers with different lactide–glycolide ratios differ in their hydrophilicity and interactions with the drug, resulting in different loading and release profiles. The crystallinity of PLGA is influenced by the stereochemistry of the lactic acids. L-PLA and PGA are semi-crystalline, while D,L-PLA and PGA form an amorphous copolymer. D,L-PLGA is preferable in microsphere formulations, as it allows a more homogeneous distribution of the active ingredient. In addition, the amorphous domains are more accessible to water than the crystalline ones, so degradation proceeds more rapidly. The molecular weight of PLGA is directly related to the rate of degradation of the polymer: a lower molecular weight leads to a faster release. Replacing acid-terminated PLGA with ester-terminated PLGA while maintaining the same monomer ratio and molecular weight significantly prolongs polymer degradation due to lower hydrophilicity. Acid-terminated PLGA has a strong tendency to adsorb water and swell the polymer matrix [3].

The drug can be released from PLGA microparticles via transport through water-filled pores, transport through the polymer matrix or the degradation of the encapsulating polymer. The most common route of peptide release is transport through water-filled pores, as these molecules are too large and too hydrophilic to be transported through the polymer matrix [13,14]. The most commonly used mechanistic model for controlled-release studies is the Peppas model (also known as the power law). This model has been successfully used to describe drug transport from different polymer systems by Fickian diffusion and anomalous transport [15,16,17,18,19,20].

The aim of this work was to prepare PLGA microparticles loaded with vancomycin by using an emulsion/spray-drying technique. Vancomycin is a large hydrophilic molecule that is usually administered as an intravenous infusion for systemic therapy in high doses for 1 to more than 8 weeks, depending on the indication [21]. Methods have been described to incorporate vancomycin into extended-release systems for local treatment [22,23,24,25,26].

A high concentration of vancomycin hydrochloride in the aqueous phase of the emulsion (125 mg/g) was chosen to increase the drug-to-polymer ratio in the final dry product. However, the gelling point that occurs with highly concentrated vancomycin hydrochloride solutions was not reached. Acid-terminated D,L-PLGA with a low Mw and L/G ratio of 50/50 was used as the encapsulating polymer. The drug-to-polymer ratio was the same for all formulations tested, although the co-encapsulating excipients used (chitosan, poloxamer 407 and sorbitan monooleate in fixed concentrations) differed. The aim was to select a PLGA grade with low viscosity to allow the controlled delivery of vancomycin in accordance with the usual treatment duration. Since vancomycin is a positively charged molecule, there is a strong tendency for electrostatic interactions with the negatively charged chains of acid-terminated PLGA. This effect contributes to the stabilisation of a W/O emulsion [27] and can also influence the encapsulation and release of the active ingredient.

The operating conditions for spray drying with an ultrasonic atomiser nozzle were selected based on preliminary studies of processing PLGA-based emulsions. In order to investigate the effect of the encapsulated excipients, the microparticles were characterised in terms of their thermal behaviour, physical properties, drug loading and in vitro release. In addition, a theoretical investigation of the mechanism controlling the release of the drug from the obtained delivery systems was carried out.

## 2. Materials and Methods

### 2.1. Materials

Vancomycin hydrochloride (VAN) was purchased from Guangzhou Greensyn Pharma Co, Ltd. (Guangzhou, China). Evonik (Darmstadt, Germany) was the supplier of poly(D,L-lactide-co-glycolide) (Resomer^®^ RG 502 H, L/G ratio 50/50, acid-terminated, Mw 7000–17,000 Da, inherent viscosity 0.16–0.24 dL/g in chloroform at 25 °C). Chitosan (from shrimp shells, low viscosity (20–200 mPas, c = 1 wt% in 1% acetic acid)) was purchased from Sigma-Aldrich (Steinheim, Germany). Poloxamer 407 (P407), a poly(ethylene oxide)-poly(propylene oxide)-poly(ethylene oxide) (PEO-PPO-PEO) triblock copolymer, was purchased from BASF (Ludwigshafen, Germany). Sorbitan monooleate (Span™ 80) was purchased from Croda (Barcelona, Spain). Dichloromethane (DCM) was purchased from Merck (Darmstadt, Germany). Other materials were of analytical grade.

### 2.2. Preparation of VAN-Loaded PLGA Microparticles

PLGA microparticles loaded with VAN were prepared by emulsion/spray drying. W/O emulsion formulations were prepared by dissolving VAN (12.5% *w*/*w*) in purified water and PLGA (5% *w*/*w*) in DCM, as previously described [27]. The ratio between the dispersed aqueous phase and the continuous organic phase was 1:10 for all emulsion formulations. One of the four emulsions prepared did not contain any additives. The second emulsion prepared contained P407 (1% *w*/*w*) together with VAN in the aqueous phase. In the third emulsion, chitosan (1% *w*/*w*) was dissolved with VAN in the aqueous phase, and sorbitan monooleate (0.2% *w*/*w*) was dissolved with PLGA in the organic phase, while the fourth emulsion contained chitosan (1% *w*/*w*) dissolved together with VAN in the aqueous phase. The compositions of the emulsions are summarised in Table 1.

The emulsions were prepared by ultrasonication using a Sonopuls HD 4200 with titanium flat tip TT213 (BANDELIN electronic GmbH & Co. KG, Berlin, Germany) for 30 s at 100% amplitude in a cold bath [27]. The emulsions were spray-dried immediately after their preparation using the ProCepT 4M8-TRIX spray dryer (Procept, Zele, Belgium). The spray dryer was equipped with a 25 kHz ultrasonic nozzle and was cooled with compressed air during the process. The emulsions were kept at room temperature during drying with constant stirring. The emulsions were transferred from a beaker through a set of flexible (1.02 mm/41 cm) and non-flexible tubing (1.8 mm/115 cm) to the atomisation chamber using a peristaltic pump. The process parameters were as follows: air inlet temperature 80 °C, air inlet flow rate 0.34 m^3^/min, liquid flow rate 1.5 mL/min (pump rate 25%) and differential pressure across the cyclone 25 mBar. An air outlet temperature of 38–40 °C was achieved.

The solid powder product (VAN-loaded PLGA microparticles) was removed from the collection container of the spray dryer, immediately transferred to a storage bottle and purged with nitrogen before sealing. The sealed bottles were stored protected from light and refrigerated (2–8 °C). Analyses were performed on freshly prepared samples within a few days of spray drying.

The powder yield (%) of the spray-drying process was calculated as the percentage by weight of the solid originally contained in the volume of the atomised emulsion that could be recovered from the collecting vessel. The powder located on the inner wall of the cyclone was not considered part of the yield.

### 2.3. Characterisation of VAN-Loaded PLGA Microparticles

#### 2.3.1. VAN Loading and Encapsulation Efficiency

For the determination of drug loading (DL) and encapsulation efficiency (EE), about 3 mg of microparticles was accurately weighed, dissolved in 2 mL of tetrahydrofuran and 10 mL of highly purified water, diluted to 20 mL with highly purified water and centrifuged at 5000 rpm for 15 min. The supernatant was filtered through a 0.2 µm filter (RC) and analysed by high-performance liquid chromatography (HPLC). The HPLC system consisted of an Agilent 1100 series instrument (Agilent Technologies, Santa Clara, CA, USA) with a diode array detector at 240 nm and a column (Kinetex C18 column 50 × 4.6 mm^2^, particle size 2.6 μm, Phenomenex, Torrance, CA, USA) with an inline filter (KrudKatcher Ultra HPLC, 0.5 µm depth filter × 0.004 inch, Phenomenex, Torrance, CA, USA). The mobile phase was acetonitrile–water–trifluoroacetic acid (15:85:0.1%, V/V/V). The analysis was carried out under the following chromatographic conditions: a mobile phase flow rate of 1 mL/min, a temperature of 45 °C and a sample injection volume of 50 μL. The elution was isocratic, and the run time was 1 min.

The drug loading (DL) was calculated as the mass percentage ratio (% *w*/*w*) of the determined VAN content in the investigated quantity of microparticles.

The encapsulation efficiency (EE) was calculated as the mass percentage ratio (% *w*/*w*) between the determined VAN content (DL) and the theoretical VAN content in the investigated quantity of microparticles.

In addition, the rinsed (non-encapsulated) VAN fraction (RF) was determined by rinsing about 20 mg of accurately weighed microparticles with 20 mL of highly purified water. The VAN concentration in the water fraction obtained after filtration of the microparticle suspension through a 0.2 µm filter (RC) was determined by the HPLC method described above.

The amount of entrapped VAN (*w*/*w*%) was calculated from the difference between the drug loading (DL) (% *w*/*w*) and the rinsed fraction (RF) (% *w*/*w*).

#### 2.3.2. Particle Size Distribution

The particle size distribution was analysed by laser diffraction using a Mastersizer 3000 (Malvern Panalytical Ltd., Worcestershire, UK). About 100 mg of the sample was placed in a beaker and manually dispersed with a glass rod in about 1.5 mL of a 0.5% PVA solution. This suspension was added dropwise to the dispersing unit of the instrument until a turbidity between 2.0% and 8.0% was achieved. The measurement was carried out in water with 40% ultrasound for 75 s at a stirrer speed of 2000 rpm. Both red and blue light measurements were taken, each lasting 60 s. The microparticle size was expressed as *D*_4,3_ $\left(D_{\left[4,3\right]}=\sum nd^{4}/\sum nd^{3}\right)$, where *d* is the particle diameter and *n* is the number of particles. Span, a parameter related to the width of a distribution, was expressed as Span = ((d90% − d10%)/d50%), where d10%, d50% and d90% are the equivalent volume diameters at 10, 50 and 90% of the cumulative volume, respectively. Three consecutive measurements were taken for each aliquot, excluding those that showed interference (air bubbles).

#### 2.3.3. Morphology

Powder samples were fixed to aluminium plates with conductive double-sided adhesive tape. Excess particles were detached by directing a dry air jet onto the surface of the platelets. The samples were coated with gold in an Edwards S150 sputter coater, and the morphology of the microparticles was examined using a Jeol JSM-5900 scanning electron microscope LV (Jeol, Tokyo, Japan) at an accelerating voltage of 10 kV.

#### 2.3.4. Powder X-ray Diffraction (XRD)

Powder X-ray diffraction (XRD) patterns of the pure drug, pure polymer and microparticles were obtained using an X’Pert Pro X-ray diffractometer equipped with an X’Celerator detector (Malvern Panalytical Ltd., Worcestershire, UK). Diffraction data were acquired by exposing powder samples to Cu-Kα X-ray radiation (1.5418 Å) using 45 kV voltage and a current of 40 mA. Samples were loaded on a silicon zero-background holder and scanned from 3 to 40° 2Theta. The total scanning time was 0.5 h per sample (with a step size of 0.017° 2Theta and a scan step time of 100 s).

#### 2.3.5. Infrared Spectra (FT-IR)

The infrared spectra (IR) were recorded with a PerkinElmer Frontier IR Dual-Range spectrometer (650–4000 cm^−1^) using the attenuated total reflection (ATR) technique (PerkinElmer, Shelton, CT, USA). Sample analyses were performed using PerkinElmer Spectrum software IR ES version 10.6.0. Infrared spectra of the pure drug (VAN), the pure polymer (PLGA), the prepared microparticles (M1–M4) and the corresponding physical mixtures (PM1–PM4) were recorded.

In order to evaluate the structure of encapsulated VAN, samples of VAN extracted from the microparticles were prepared. An 80 mg sample of microparticles was incubated with 1.5 mL of water for 24 h at 2–8 °C and centrifuged (2 min at 14,000 rpm). The supernatant was filtered through a 0.8 µm syringe filter into a clean glass vial and freeze-dried with an SP VirTis AdVantage Pro freeze dryer (SP Scientific, Ipswich, UK) using the following process parameters: freezing for 3.5 h at −40 °C; drying for 8.5 h at −25 °C, 10.8 h at 0 °C, and 4.4 h at 10 °C under vacuum (200 µbar). Freeze-dried samples of VAN extracted from the microparticles (M1(lyo)–M4(lyo)) were analysed as well.

#### 2.3.6. Differential Scanning Calorimetry (DSC)

DSC of the pure drug was measured using a DSC Discovery differential scanning calorimeter (TA Instruments, New Castle, DE, USA). Approximately 3.5 mg of the sample was weighed into a standard aluminium dish. The sample was equilibrated at 20 °C for 5 min and heated to 200 °C (ramp 5 °C/min) by using a modulated temperature amplitude of 1 °C every 60 s.

The thermal properties of the pure polymer, the prepared microparticles and the corresponding physical mixtures were measured using the DSC 2000 differential scanning calorimeter (TA Instruments, New Castle, DE, USA). Approximately 0.6–1.6 mg of each sample was weighed into an aluminium dish, which was then sealed, and a tiny pinhole was made to allow excess vapour to escape. The sample was equilibrated at 20 °C, heated to 120 °C (ramp 10 °C/min), then cooled to −20 °C and reheated to 150 °C. The first heating step was performed to quench the thermal evolution of the polymer and to obtain the glass transition temperature in the second stage.

#### 2.3.7. In Vitro Release Testing of VAN-Loaded PLGA Microparticles

The in vitro release experiments were performed according to the method developed by Lusina Kregar et al., 2015 [28], with slight modifications. A VK7010 dissolution apparatus with 200 mL flat-bottom vessels and mini-paddles (Agilent Technologies) was used. Immersion cells (Enhancer Cell^TM^, Agilent Technologies, Santa Clara, CA, USA) with a surface area of 4 cm^2^ were used as sample compartments. A total of 50 mL of phosphate buffer (PBS) pH 7.4 at a temperature of 37.0 ± 0.5 °C was used as the release medium. Hydrophilic polypropylene membranes with a thickness of 0.45 µm (PALL) were used for the release tests. The membranes were soaked in the release medium for 30 min before starting the test. The volume of the sample compartment was set to 1 mL, and an appropriate amount of the sample containing 5 mg of VAN was added to the sample compartment. The remaining volume of the cell cavity was filled with the release medium. The contents of the sample compartment were carefully mixed with a spatula. The membrane was then placed over the sample compartment, sealed, and secured with a washer and retaining ring. The assembled cell was placed in the dissolution vessel with the membrane facing upwards. The rotation speed of the paddle was 50 rpm. The height of the paddle was set to 1 cm above the membrane. Samples (2 mL) were taken at time points of 0.5, 2, 4, 6, 24, 48, 72, and 96 h and replaced with an equal volume of thermostated release medium. The samples were analysed by the HPLC method described previously. The cumulative dilution caused by replacing the sample with fresh release medium was corrected. Sink conditions were kept throughout the experiment.

To determine the amount of VAN that was not released during the study, residual samples from the sample compartments were carefully transferred to 50 mL volumetric flasks. The dip cells and membranes were rinsed with 5 mL of tetrahydrofuran in the flask, and 20 mL of highly purified water was added to dissolve the remaining microparticles. The samples were then diluted to 50 mL with highly purified water and centrifuged at 5000 rpm for 15 min. The supernatant was filtered through a 0.2 µm filter (RC) and analysed by the HPLC method.

For the analysis of the release mechanism, Peppas’ equation (Equation (1)) was used [15,16]:

(1) $$X_{t}/X_{inf}=kt^{n}$$

where *k* is the release rate constant, *n* is the release exponent and $X_{t}/X_{inf}$ is the fraction of the drug released at time *t*. Depending on the geometry of the swellable controlled-release system (thin film, cylinder or sphere), the value of the obtained release exponent *n* indicates the mechanism of drug release.

### 2.4. Statistical Evaluation

All results are expressed as mean ± standard deviation (SD). Statistical analysis between two groups was performed using Student’s *t*-test, and multiple groups were assessed using the one-way ANOVA test followed by Dunnet’s post hoc test. A value of *p* ≤ 0.05 was considered statistically significant.

## 3. Results

### 3.1. Encapsulation Efficiency and Drug Loading

The properties of the spray-dried VAN-loaded PLGA microparticles, M1–M4, are shown in Table 2. All values are expressed as mean ± SD, *n* = 4, except for entrapped VAN, which was calculated from the difference between mean values obtained for DL and RF.

The results show high encapsulation efficiency, with similar values obtained for formulations M1–M4 (*p* = 0.2437). However, statistically significant differences (*p* ˂ 0.0001) are observed for the rinsed and consequently entrapped VAN. The rinsed VAN fraction is very low for M1 (without additives) and M4 (with chitosan), with similar values obtained for both formulations (*p* = 0.57). M2 (with P407) and M3 (with chitosan and sorbitan monooleate) have significantly higher amounts of the rinsed drug compared to the aforementioned formulations (*p* ˂ 0.0001).

### 3.2. Particle Size Distribution

Table 3 shows the volume-based particle size distribution (d10%, d50%, d90%, *D*(4,3), Span) for spray-dried microparticles M1–M4.

The particle size data show that the microparticles produced were micron-sized. Sample M4 (with chitosan) has the largest particle size, with statistically significant differences in d10, d50, d90 and *D*[4,3] compared to the other samples (*p* < 0.05). Samples M1, M2 and M3 do not show a significant difference in these particle size distribution parameters. The difference in the Span value for M1 and M4 is not statistically significant (*p* = 0.0698). The Span value is higher for M2 than for other samples, while M3 has the lowest Span.

### 3.3. Morphology

**Figure 1:**
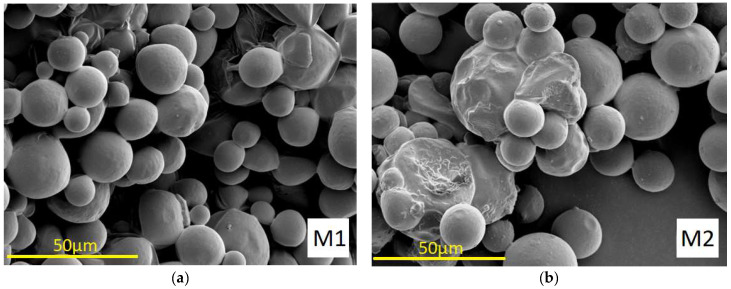

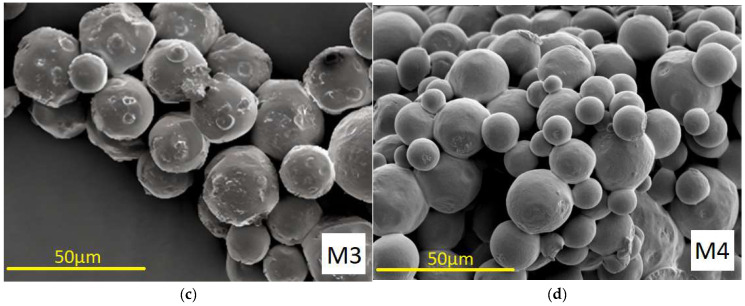
SEM images (M = 1000×) of VAN-loaded PLGA microparticles: (**a**) M1; (**b**) M2; (**c**) M3; (**d**) M4.

The morphology of PLGA microparticles M1–M4 loaded with VAN produced by emulsion spray drying is shown in Figure 1.

As the images from SEM (Figure 1) show, the spray-dried microparticles M1 (without additives), M2 (with P407) and M4 (with chitosan) are spherical with smooth, non-porous surfaces. The morphology of microparticles M3 (with chitosan and sorbitan monooleate) has a different surface pattern with many craters. It can also be observed that certain particles are partially fused together, especially in sample M2.

### 3.4. X-ray Powder Diffraction

**Figure 2:**
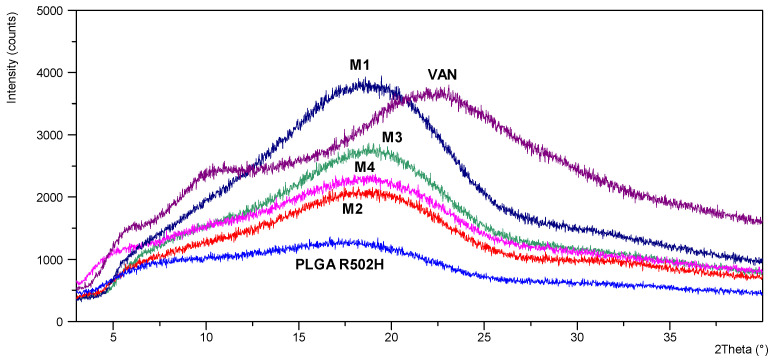
XRD patterns obtained from VAN (purple), PLGA R502H (blue) and VAN-loaded PLGA microparticles M1 (dark blue), M2 (red), M3 (green), M4 (pink).

X-ray powder diffraction of pure vancomycin hydrochloride shows that the material is amorphous, as described by Kaduk et al. (2014) [29]. Comparison of the X-ray diffraction patterns of VAN, PLGA polymer and VAN-loaded PLGA microparticles showed no significant differences, indicating that the amorphous nature of vancomycin was not affected by the microencapsulation process (Figure 2).

### 3.5. The Compatibility Study

The compatibility between the polymer and the drug in VAN-loaded PLGA microparticles was determined by FT-IR and DSC. Both analyses were performed for the pure drug substance (VAN), the pure PLGA polymer, the VAN-loaded microparticles (M1–M4) and the physical mixtures with identical proportions of the microparticle components to evaluate possible chemical interactions between the components of the formulation and the drug. In addition, the structure of VAN, which was extracted from each of the four microparticle formulations, was evaluated using the FT-IR technique.

#### 3.5.1. Infrared Spectra (FT-IR)

**Figure 3:**
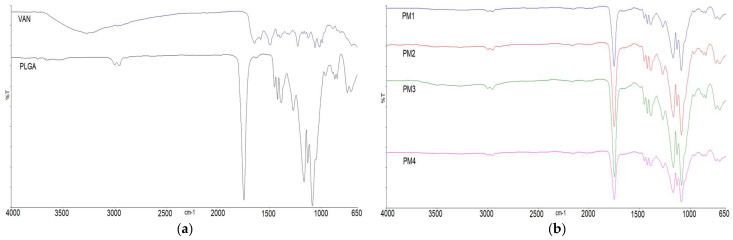

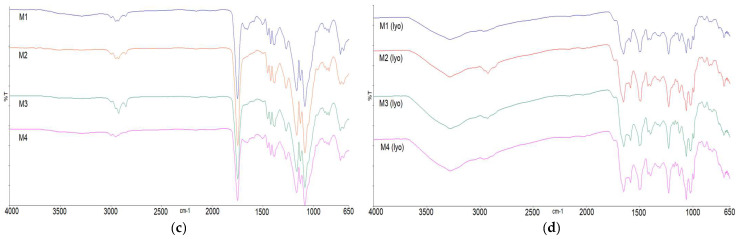
IR spectra of: (**a**) pure VAN and pure PLGA; (**b**) physical mixtures that correspond to formulations M1–M4 (PM1–PM4); (**c**) spray dried microparticles M1–M4; (**d**) VAN extracted from microparticles M1–M4 to the aqueous media and freeze dried (M1 (lyo)–M4 (lyo)).

The infrared spectrum of pure VAN (Figure 3a) showed hydroxyl stretching at 3257 cm^−1^, amide C=O stretching at 1646 cm^−1^, amide N-H bending at 1586 cm^−1^, aromatic C=C stretching at 1505 cm^−1^ and phenolic C-O stretching at 1227 cm^−1^. The absorption peaks in the range between 1350 cm^−1^ and 900 cm^−1^ correspond to C-O and C-N stretching vibrations. The characteristic absorption peaks in the IR spectrum of the pure PLGA polymer (Figure 3a) showed C-H stretching at 2950 cm^−1^, ester C=O stretching at 1748 cm^−1^, C-H bending in the range of 1460–1300 cm^−1^ and C-O stretching in the range of 1300 to 1000 cm^−1^ [23]. The FT-IR spectra of the physical mixtures (PM1–PM4) (Figure 3b) and PLGA microparticles loaded with VAN (M1–M4) (Figure 3c) contain mostly visible peaks of the polymer. Absorption peaks attributed to the presence of VAN in M1–M4 microparticles were barely visible in the range 1650–1500 (C=O stretching of the amide and N-H bending), similar to the four formulations. Other peaks characteristic of VAN were not visible, as they overlapped with the strong absorption peak of the C-O stretching of the polymer. To better elucidate the structure of the encapsulated drug, VAN was extracted from the microparticles in the aqueous medium and freeze-dried. The infrared spectra of the prepared samples (M1–M4 lyo) (Figure 3d) show absorption peaks characteristic of VAN, since the polymer has been removed by extraction. There are slight differences between the four formulations.

#### 3.5.2. DSC

**Figure 4:**
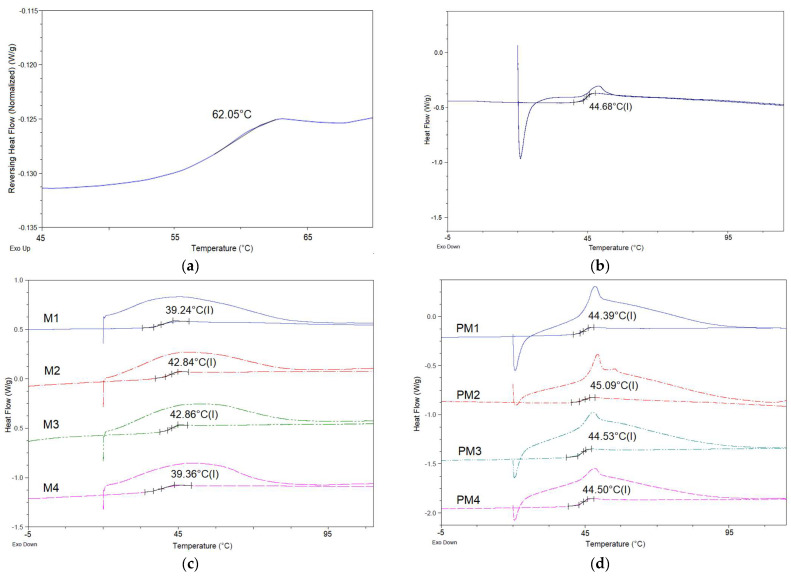
Thermograms of (**a**) pure VAN; (**b**) PLGA Resomer R502H; (**c**) spray-dried VAN-loaded microparticles M1–M4; (**d**) physical mixtures that correspond to M1–M4.

DSC studies were performed on the following samples: pure polymer and VAN (Figure 4a), VAN-loaded PLGA microparticles M1–M4 (Figure 4b) and their corresponding physical mixtures containing the same substances as the microparticle formulations (Figure 4c). The baseline shift in the thermogram of the pure VAN compound with a midpoint at 62 °C (Figure 4a) corresponds to a phase transition, which is characteristic of the amorphous drug [30]. Resomer 502H has a Tg value of 44.68 °C. In the DSC thermograms of the microparticles loaded with VAN (Figure 4c) and their physical mixtures (Figure 4d), endothermic events related to the dehydration of vancomycin and the glass transition temperature of the polymer were observed, with no other events detected.

### 3.6. In Vitro Release Study

**Figure 5:**
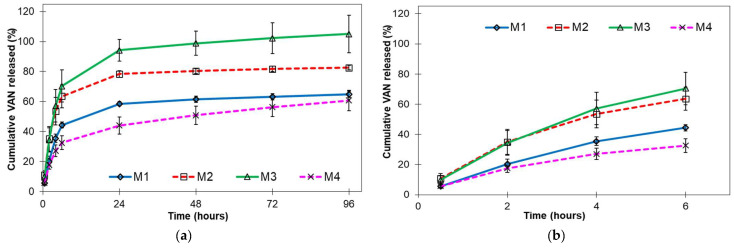
In vitro release profiles of VAN-loaded PLGA microparticles: (**a**) 96 h release; (**b**) initial release up to 6 h (mean ± SE, *n* = 3).

Figure 5 shows the cumulative in vitro release profiles of VAN-loaded PLGA microparticles.

A Peppas analysis was performed by plotting the logarithm of the percentage of drug released and the logarithm of time [31]. A profile consisting of two linear phases was obtained for each formulation, with the slope of each linear phase equal to the release exponent *n* (Table 4).

## 4. Discussion

The physicochemical properties of microparticles can influence the release rate, in vivo distribution, side effect profile and injectability of the parenteral dosage form.

The polymeric microparticles loaded with VAN prepared by spray drying the described W/O emulsions exhibited high encapsulation efficiencies (78.0–88.2% *w*/*w*), with similar values when comparing the different formulations (M1–M4), as shown in Table 2. However, the internal structure, and in particular the distribution of VAN within the polymer matrix of a microparticle, is influenced by the presence and nature of the additive in the formulation. As soon as the microdroplets of a W/O emulsion enter the drying chamber, there is solvent removal and solute migration through the drying droplets, which depends on the composition of the whole system. This affects the distribution of solutes in the resulting spray-dried particles and the encapsulation efficiency [32].

The total drug loading of spray-dried PLGA microparticles (M1–M4) is represented by two main fractions: the fraction of VAN that remains on or near the surface of the particles and the fraction of VAN that is encapsulated in the polymer matrix of the particles. The first fraction of VAN can be easily washed out, as VAN is very soluble in water. The second VAN fraction is located inside the microparticles and/or is partially bound to the surface via electrostatic interactions [33].

The rinsed VAN fraction is very low at 0.9% and 1.2% in M1 (without additives) and M4 (with chitosan), respectively, suggesting that the drug is mainly distributed in the polymer matrix of the particles. This could be due to ionic interactions between the negatively charged carboxyl group of the PLGA polymer and the positively charged amino acid groups of the glycopeptide VAN, which enhance drug retention [34,35]. Due to the diversity of pKa values, VAN contains both positive and negative charges. The addition of chitosan affects the electrostatic interactions between VAN and PLGA. Positively charged groups of chitosan could interact with the negatively charged carboxyl group of VAN and thus increase the drug loading. However, the acidic end of PLGA may consume some of the positively charged groups of chitosan available for interaction with VAN [23]. In addition to the electrostatic effect, the large hydrophilic chains of chitosan provide steric shielding that enhances VAN retention. The resulting encapsulation efficiency for the formulation with chitosan (M4) is very similar to that without additives (M1). The presence of sorbitan monooleate in the formulation (M3) causes the molecules of VAN to be displaced from the W/O interface and migrate closer to the surface of the forming microparticles, despite the electrostatic and steric effects of chitosan. In the case of P407, microparticles M2, the incorporation of VAN is higher at 12.2% than microparticles M3 at 7.1%. The reason for this could be the polymeric nature of P407, which prevents the migration of the drug.

The microstructure of the feed emulsion is another important aspect that can influence the encapsulation efficiency. The flow conditions in the feed tubes lead to some shear stress and pose a risk to the stability of the microstructure of the emulsion. This depends on the dimensions of the tubes as well as the emulsion properties. If the shear stress during emulsion flow is too high, there is a risk that the droplets of the dispersed phase will break apart, affecting the distribution of the drug in the polymer matrix of the dry microparticles.

The significantly higher amounts of VAN on the surfaces of microparticles M2 and M3 compared to M1 and M4 can also be explained by the microstructure of the feed emulsion before spray drying. Namely, the feed emulsions used in the preparation of microparticles M2 and M3 had lower values of zero-shear viscosity and viscosity decrease as a function of shear stress than the emulsions used in the preparation of M1 and M4 [27]. Due to the different ability of the emulsions to withstand the applied shear stress, it is possible that droplet disruption occurred during flow, which eventually resulted in a significant amount of VAN on the surface of the spray-dried microparticles. In contrast, the emulsions used to produce M1 and M4 microparticles were able to resist the structural changes during flow, resulting in the excellent encapsulation of VAN in the polymer matrix.

As the images from SEM (Figure 1) show, spray-dried microparticles M1, M2 and M4 are spherical with smooth, non-porous surfaces. The co-encapsulation of sorbitan monooleate (M3) had a strong effect on the morphology of the microparticles, as evidenced by a different surface pattern with many craters. This could be due to the displacement of VAN from the W/O interface to the outer surface of the drying droplet due to the influence of a surfactant present in the formulation, sorbitan monooleate. It can be seen (Figure 1) that certain particles have partially fused together, especially in the case of the M2 microparticles. The probable cause of this is the initial temperature of spray drying (45 °C), which was close to the glass transition temperature of PLGA, resulting in the stickiness of the microparticles and their agglomeration [36]. The migration of additives into the organic phase during emulsification can also promote the agglomeration of the microparticles [37,38].

The spray-drying yield ranges from 18% for M2 to 45% for M4 and is quite low, with visible product deposits on the spray chamber walls. The same liquid flow rate of 1.5 mL/min (pump rate 25%) was set for all experiments, so the differences in the production yield are due to the different qualitative compositions of the individual samples. The lowest yield was obtained with P407 as an additive in the dispersed phase of the emulsion (M2). This could be due to the fact that P407 migrates from the aqueous to the organic phase, leading to the aggregation of the microparticles [37]. On the other hand, the co-encapsulation of chitosan significantly increased the yield of the dry product (sample M4) and was twice as high as the yield of sample M2. Chitosan is a charged molecule, and its presence in the formulation can alter the surface charge of the dry microparticles, reducing their tendency to aggregate and consequently increasing the process yield. It is evident that the choice of excipients in the spray-drying process can have a significant impact on processability, along with the effects on product quality attributes.

To achieve adequate drug release, ensure acceptable injectability and avoid immune reactions, polymeric microparticles with a diameter of 10 to 200 μm are preferred [39]. Particle size data show that the microparticles produced fall within this range. In general, the viscosity is proportional to the starting fluid, while the surface tension is inversely proportional to the droplet size in the spray-drying atomisation process and, consequently, to the particle size of the final dry product. The situation is different in ultrasonic atomisation, where a higher viscosity of the feed liquid can lead to the formation of smaller droplets. Due to the higher viscosity, the dwell time of the liquid on the vibrating plate is longer. Within this time, there is a local temperature increase due to energy dissipation from the vibration, resulting in a decrease in fluid viscosity and the formation of smaller droplets [40,41]. As described in Juric Simcic et al., 2023 [27], W/O emulsions containing vancomycin and PLGA (corresponding to samples M1–M4) show non-Newtonian shear thinning behaviour. Compared to the other formulations, the chitosan-containing emulsion (corresponding to M4) shows the highest zero-shear viscosity and a slightly higher viscosity in the low shear rate range (up to 1 s^−1^), which is due to a narrower droplet size distribution. The viscosity values decrease with increasing shear stress, so the viscosity curves of the tested emulsions converge more in the areas of higher shear stress. The apparent viscosity on the vibrating surface of an ultrasonic nozzle decreases towards higher shear rates, with all tested emulsions showing similar viscosity. Microparticles with encapsulated chitosan (M4) have a larger particle size than other samples, indicating that the size of the atomised droplets was larger. Since the operating conditions were the same and the apparent viscosity on a vibrating surface was similar for all four formulations, we can assume that the presence of chitosan increased the resistance to droplet break-up due to the higher viscoelasticity of the emulsion [42], compared to emulsions without chitosan. The addition of sorbitan monooleate to the organic phase of the chitosan-containing emulsion resulted in a decrease in the mean diameter of microparticles M3 compared to M4, as well as a narrower particle size distribution (lower Span value). Due to the surface-active character of sorbitan monooleate, the interfacial tension at the air/droplet interface is lower. This reduces the effects of the viscoelasticity of chitosan, resulting in the formation of smaller droplets during atomisation. The addition of P407 to the aqueous phase of the emulsion had no significant effect on the particle size, except for the broader distribution (higher Span value) of dried microparticles M2 compared to microparticles M1 prepared from an emulsion without additives.

FT-IR and DSC were performed for the pure drug substance (VAN), the pure polymer (PLGA Resomer 502H), the VAN-loaded microparticles (M1–M4) and the physical mixtures with identical proportions of the microparticle components (PM1–PM4) to evaluate possible chemical interactions between the components of the formulation and the drug. Differential scanning calorimetry provided information on the thermal transformations of the dry polymeric microparticles. The data collected were compared to determine the nature of the interactions between the constituents of the microparticles and whether they form some kind of physical or chemical bond. As there was no significant change in the glass transition of PLGA, this indicates the absence of a detectable plasticising effect and thus the absence of an interaction between the polymer and the drug. Furthermore, the single inflection in the DSC thermograms proves the miscibility of the polymers PLGA and chitosan and the amorphous form of the drug. The amorphous nature of pure VAN and VAN encapsulated in formulations M1-M4 was also confirmed by powder X-ray diffraction analysis.

No differences in the positions of the absorption bands of VAN were observed in the FT-IR spectra of the prepared physical mixtures PM1–PM4 or microparticles M1–M4, indicating that there are no chemical interactions between the drug and the polymer in the solid state (Figure 3) [23,43]. The co-encapsulation of P407, chitosan and/or sorbitan monooleate resulted in no detectable changes in the position or intensity of the VAN-specific absorption peaks, indicating that there is no interaction between the drug and excipients. In general, electrostatic interactions between the oppositely charged groups of the formulation ingredients are not observed in the solid state.

However, most of the peaks characteristic of VAN were not visible in the physical mixture and microparticle samples because they overlapped with the strong absorption peaks of the PLGA polymer. To better elucidate the structure of the encapsulated drug, VAN was extracted from the microparticles in the aqueous medium, freeze-dried and also recorded. The infrared spectra of the prepared samples (M1–M4 lyo) contain absorption peaks characteristic of VAN, as the influence of the polymer absorption bands is largely excluded. Interestingly, the peaks at 1395 cm^−1^ and 1175 cm^−1^, characteristic of VAN, are barely seen in M1 (lyo; without additive) and M2 (lyo; with P407), in contrast to samples M3 (with chitosan/Span 80) and M4 (with chitosan), suggesting that there may be structural differences between the encapsulated VAN in different samples. The structural integrity and antimicrobial efficacy of vancomycin released from microparticles need to be further investigated to determine the potential protective effects of specific formulation ingredients and to gain a full understanding of the impact that a particular formulation additive may have on the quality of the final drug product.

The cumulative in vitro release profiles (Figure 5) show that prolonged release of VAN was achieved by encapsulating VAN in PLGA microparticles. All formulations show a biphasic release profile with a rapid phase I release (burst release) and an apparent zero-order release during phase II.

Microparticles prepared from the emulsions with surfactants P407 (M2) and sorbitan monooleate (M3) released the VAN most rapidly and completely. As shown in Figure 5, the release was complete after one day for the M3 microparticles. This is consistent with the other results obtained for the M3 microparticle sample: the highest result for rinsed VAN (Section 3.1.), the smaller mean particle diameter (Section 3.2.) and the higher porosity of the microparticles (Section 3.3.) compared to other microparticle samples. All results indicate that a large proportion of VAN was distributed on or near the surfaces of the microparticles, which is known to lead to a high release, as the drug molecules are easily accessible due to hydration near the surface. Moreover, the release would be faster due to the disturbed surface [13]. Surfactants can interact with drug molecules and disrupt drug–polymer interactions, resulting in a higher amount of free drug and faster release compared to formulations without surfactants [34]. The burst release may also be related to the spray-drying process. The migration of the active ingredient during the drying process leads to the heterogeneous distribution of the active ingredient and facilitates the burst release [44]. Microparticles prepared from a P407-containing emulsion (M2) achieve a similar initial VAN release to M3 microparticles. Similar to sorbitan monooleate, P407 increases the hydrophilicity of the polymer matrix and thus the water uptake. Due to its solubilising and wetting effects, P407 is known to greatly increase the release of drugs [9]. After the initial release, microparticles containing P407 (M2) show a slower release compared to microparticles containing sorbitan monooleate (M3) due to more efficient VAN encapsulation.

The release of VAN from microparticles prepared from an emulsion without additives (M1) or with chitosan only (M4) was significantly lower than the release from microparticles M2 and M3. After four days, a cumulative release of 65% and 61% was achieved for microparticles M1 and M4, respectively. The release due to bursting was also lower, especially for microparticles with chitosan (M4). This is consistent with the high VAN inclusion (Section 3.1) and the highest mean particle diameter (Section 3.2).

All of the unreleased VAN was in the immersion cell compartment, as was the polymer residue. It appears that microparticles prepared from emulsions without surfactants (M1 and M4) aggregate more readily than microparticles prepared from emulsions with surfactants (M2 and M3), limiting the release of VAN.

Since, on average, the highest amount of the drug was released during the first phase, this phase is considered the most interesting and suitable for further modelling. The second phase is also included in the modelling to obtain additional information.

A Peppas analysis was performed by plotting the logarithm of the percentage of drug released and the logarithm of time [31]. A profile consisting of two linear phases was obtained for each formulation, with the slope of each linear phase equal to the release exponent *n* (Table 4).

For spherical systems with controlled release, the release exponent *n* = 0.43 corresponds to Fickian diffusion, 0.43 < *n* < 0.85 corresponds to anomalous (non-Fickian) transport, *n* = 0.85 corresponds to case II transport defined by polymer swelling and drug release controlled by relaxation and diffusion, and *n* > 0.85 corresponds to super case II transport [15,16,18]. This model was created for a monodisperse system (microparticles of a certain size). For polydisperse spherical microparticles, *n* values around 0.3 ± 0.1 are also indicative of Fickian diffusion [16]. Also, in PLGA-based systems, where drug release occurs by diffusion through the polymer network, the release exponent *n* shifts to smaller values as a result of combined diffusion through a swollen matrix and through water-filled pores [31].

Assuming that the prepared microparticles (M1–M4) represent spherical delivery systems, the obtained *n* values (Table 4) show that phase I of the release of VAN from microparticles prepared from an emulsion without additives (M1) is generally described as case II transport due to polymer swelling (*n* = 0.85). In contrast, the initial phase of the release of VAN from all other samples (M2–M4) is dominated by non-Fickian diffusion. However, the methodology used for the in vitro release assay should also be considered when evaluating the mechanism of drug release. The microparticles were dispersed in an immersion cell and separated from the release medium by a membrane, so they were subjected to limited movement. Under the conditions described, polymeric microparticles tend to “stick” to each other and aggregate, so the system tends to have a thin-film geometry. In this case, the predominant mechanism for M1 microparticles would also be anomalous transport (non-Fickian diffusion). Nevertheless, it is evident from the release exponents obtained that polymer swelling during the first release phase appears to be more pronounced for microparticles prepared from an emulsion without additives (M1) than for the other microparticles (M2–M4). Although it is known that release in the burst phase is mainly determined by the dissolution of the immediately available drug on the surface of the microparticles and diffusion through the initially present pores [13], the mechanism of polymer swelling during the burst release has already been described in the modelling of in vivo data on peptide release from PLGA microparticles [31].

In phase II, the release of VAN from all microparticles is subject to the same mechanism, i.e., the release of VAN is dominated by Fick’s diffusion (*n* = 0.1–0.2), suggesting that the release of the drug in this last phase is determined by diffusion through water-filled pores in the polymer matrix.

## 5. Conclusions

The results show that the W/O emulsion/spray-drying method is generally suitable for the preparation of VAN-loaded PLGA microparticles with high drug loading. While the thermal and spectral properties were similar for all four formulations, the absence or presence of emulsion stabilisers altered the morphology, particle size, encapsulation efficiency and release rate of vancomycin. Furthermore, the results of the release tests using the mechanistic Peppas model indicate that diffusion is the primary mechanism for the release of VAN from all formulations.

The emulsions with chitosan in the aqueous phase were able to resist structural changes during flow, resulting in the excellent encapsulation of VAN in the polymer matrix and its prolonged release. In addition, the presence of chitosan seems to allow the structural maintenance of the VAN molecule within the PLGA matrix, as shown by the FT-IR studies of lyophilised VAN extracts from microparticles. However, further studies on the structural integrity and antimicrobial efficacy of encapsulated vancomycin are needed to determine the potential protective effect of specific formulation ingredients and to gain a full understanding of the impact that a particular formulation additive may have on the quality of the final drug product.

## Figures and Tables

**Table 1:** The compositions of emulsions.

Emulsion	Aqueous Phase (% *w*/*w*), 5 g			Organic Phase (% *w*/*w*), 50 g	
	Vancomycin Hydrochloride	P407	Chitosan	PLGA	Sorbitan Monooleate
1	12.5	-	-	5	-
2	12.5	1	-	5	-
3	12.5	-	1	5	0.2
4	12.5	-	1	5	-

**Table 2:** VAN loading and encapsulation efficiency of M1–M4 PLGA microparticles.

PLGA Microparticle Sample	M1	M2	M3	M4
Encapsulation efficiency, EE, (% *w*/*w*)	87.8 ± 9.5	88.2 ± 6.5	78.0 ± 3.3	85.7 ± 9.1
Drug loading, DL, (% *w*/*w*)	17.1 ± 1.9	16.8 ± 1.3	14.5 ± 0.6	16.5 ± 1.7
Rinsed VAN (% *w*/*w*)	0.9 ± 0.5	4.6 ± 1.0	7.4 ± 0.9	1.2 ± 0.8
Entrapped VAN (% *w*/*w*)	16.2	12.2	7.1	15.2

**Table 3:** Particle size distribution for different formulations of VAN-loaded PLGA microparticles.

	Particle Size (µm ± SD)				
Sample ID	d10%	d50%	d90%	*D*[4,3]	Span
M1	15.4 ± 0.1	36.0 ± 0.1	82.3 ± 0.6	43.2 ± 0.3	1.9 ± 0.0
M2	14.5 ± 0.0	33.9 ± 0.4	87.7 ± 1.6	43.5 ± 0.6	2.2 ± 0.0
M3	15.4 ± 0.5	36.6 ± 1.7	74.0 ± 1.0	41.0 ± 1.1	1.6 ± 0.1
M4	18.3 ± 1.7	52.9 ± 8.4	123.6 ± 20.7	64.0 ± 12.6	2.0 ± 0.1

**Table 4:** Fitted Peppas model for biphasic in vitro release—equations and coefficients of determination.

Sample ID		M1	M2	M3	M4
Phase I (0–6 h)	Equation	y = 0.8517x + 1.0187	y = 0.7234x + 1.2789	y = 0.8036x + 1.2568	y = 0.7201x + 0.9865
	R^2^	0.994	0.987	0.993	0.990
Phase II (6–96 h)	Equation	y = 0.1357x + 1.5548	y = 0.0941x + 1.7417	y = 0.1582x + 1.7504	y = 0.2225x + 1.3373
	R^2^	0.942	0.910	0.945	0.999

## Data Availability

Data are included in the article.
